# Multi-Omics and Clinical Data Analyses of Protein Arginine Methyltransferases in Pan-Cancer and Colorectal Cancer

**DOI:** 10.7150/ijms.129345

**Published:** 2026-07-13

**Authors:** Jialing Xie, Qihui Wu, Yuanyuan Xu, Jiaxin Liu, Yan Wang, Xuan Wang, Xiaodan Fu, Yimin Li

**Affiliations:** 1 Department of Pathology, Ruijin Hospital, Shanghai Jiaotong University School of Medicine, Shanghai 200025, China.; 2 Department of Gynecology, Xiangya Hospital, Central South University, Changsha 410008, China.; 3 National Clinical Research Center for Geriatric Disorders, Xiangya Hospital, Changsha 410008, China.; 4 Department of Pathology, School of Basic Medical Sciences, Central South University, Changsha 410078, China.; 5 Department of Pathology, Xiangya Hospital, Central South University, Changsha 410008, China.

**Keywords:** PRMTs, pan-cancer, colorectal cancer, genomic heterogeneity, tumor microenvironment, prognostic

## Abstract

**Objective:**

Arginine methylation, catalyzed by protein arginine methyltransferases (PRMTs), is a critical post-translational modification that modulates gene expression and signal transduction. Although the involvement of PRMTs in malignancy is increasingly recognized, a comprehensive pan-cancer synthesis of this family remains elusive. We sought to elucidate the multi-omic landscape of PRMTs and define their biological contributions to colorectal cancer (CRC) progression.

**Methods:**

Using multi-omic data from TCGA and GTEx, we performed an integrative pan-cancer analysis of PRMT expression, genetic alterations, epigenetic modifications, and their associations with prognosis, genomic heterogeneity, stemness, the tumor microenvironment, and oncogenic pathways. After validating PRMT expression patterns across multiple independent CRC cohorts from the GEO database, PRMT1 was prioritized for further investigation. Its expression was assessed in a real-world CRC cohort using immunohistochemistry, while its oncogenic potential was scrutinized through *in vitro* proliferation assays.

**Results:**

PRMT family genes exhibit heterogeneous molecular profiles across cancers, which correlate significantly with genomic instability, stemness, immune infiltration, and oncogenic pathway activation. Clinical validation demonstrated that PRMT1 is markedly upregulated in CRC, where its overexpression correlates with larger tumor size, advanced T stage, and lymph node metastasis. Loss-of-function experiments demonstrated that PRMT1 is essential for maintaining CRC cell proliferation.

**Conclusion:**

Our study highlights the multifaceted roles of PRMTs across the pan-cancer spectrum. By identifying PRMT1 as a key driver of CRC progression, this work provides a rationale for the development of PRMT-targeted therapies and personalized diagnostic biomarkers.

## Introduction

Cancer progression is driven by complex genetic and epigenetic alterations, posing persistent challenges for diagnosis and treatment 1, 2. Deciphering the epigenetic landscape across diverse malignancies is essential to identifying novel regulatory hubs 3, 4. Within this realm, protein arginine methyltransferases (PRMTs) have emerged as pivotal orchestrators of tumor biology 5. By utilizing S-adenosyl-L-methionine as a methyl donor, PRMTs catalyze the arginine methylation of diverse substrates—including histones (H3/H4) and non-histone proteins (e.g., Stat1, hnRNP)—thereby modulating fundamental cellular processes such as transcription, RNA splicing, and signal transduction 5-8. The PRMT family, comprising multiple members with distinct substrate specificities, plays an indispensable role in both physiological homeostasis and pathological progression 9-11.

Given their central role in epigenetic regulation, the dysregulation of PRMTs has been implicated in a broad spectrum of cancers; however, the precise oncogenic mechanisms underlying their action remain to be fully elucidated 12. While the overexpression or aberrant splicing of individual members, such as PRMT1, has been documented in breast, lung, and colorectal cancers (CRC) 13-16, most studies focus on isolated PRMT members or specific tumor types. This fragmented approach limits our understanding of their universal versus context-dependent functions. Although pan-cancer analyses offer a powerful tool to identify shared molecular signatures 17, existing family-wide investigations have largely been restricted to PRMT1, 4, and 5 18, 19, underscoring an urgent need for a systematic, family-wide characterization across the human cancer spectrum.

Among these malignancies, CRC remains a global health burden, ranking as the third most prevalent cancer and the second leading cause of cancer-related mortality 20, 21. Despite advances in screening, there is a critical need for novel biomarkers and therapeutic targets 22-25. Aberrant expression of PRMTs has been shown to drive CRC growth and metastasis through multiple mechanisms 26-30. Furthermore, the potent anti-tumor efficacy of emerging PRMT inhibitors in CRC models highlights their potential as both therapeutic agents and biomarkers for disease monitoring 31-33. Therefore, investigating PRMTs in CRC offers both mechanistic insights into tumorigenesis and a robust platform to validate pan-cancer findings within a clinically relevant framework.

In the present study, we integrated multi-omic data from TCGA and GTEx to provide a family-wide pan-cancer profile of PRMTs. We then focused on CRC, validating our findings using GEO datasets and a real-world clinical cohort. Through immunohistochemistry and *in vitro* assays, we specifically investigated the clinical relevance and functional role of PRMT1. Our findings provide a comprehensive landscape of PRMTs and highlight their potential as biomarkers and therapeutic targets in precision oncology.

## Materials and Methods

### Data Retrieval

To characterize the molecular and functional landscape of the PRMT family, we integrated pan-cancer transcriptomic, clinical, and multi-omic data (somatic mutations, CNVs, and DNA methylation) from TCGA and GTEx via the UCSC Xena platform. The transcriptomic signatures of PRMTs were further validated in CRC using six independent GEO cohorts (GSE9394, GSE21510, GSE32323, GSE33113, GSE39582, and GSE110224). To define their roles in clinical therapy, we further curated GEO datasets of patients receiving fluoropyrimidine-based adjuvant chemotherapy (ACT, such as FOLFOX/FOLFIRI) either alone (GSE19860, GSE28702, GSE45404, GSE62080, GSE69657, and GSE72970) or in combination with bevacizumab (GSE19860, GSE19862, and GSE72970). The single-cell RNA-seq (scRNA-seq) landscape of CRC (EMTAB8107, GSE108989, GSE112865, GSE120909, GSE122969, GSE1366397, GSE139555, GSE146771, GSE166555, and GSE179784) were obtained from the TISCH2 database (http://tisch.comp-genomics.org/home/). Furthermore, the functional necessity of PRMTs for cancer cell survival was assessed by analyzing CRISPR-Cas9 dependency scores from the DepMap portal (https://depmap.org/portal/).

### Human CRC Tissue Samples

Primary CRC specimens and paired adjacent normal tissues (≥ 5 cm from the tumor margin) were collected from treatment-naive patients at Xiangya Hospital. Inclusion required pathological confirmation of CRC and no history of neoadjuvant therapy or other malignancies. To ensure sample quality, tissues stored for over five years were excluded. A tissue microarray (TMA) consisting of 120 formalin-fixed, paraffin-embedded CRC tissues and 51 control tissues was employed for IHC analysis. Ethical approval and informed consent were obtained for the use of all clinical specimens.

### Functional Enrichment Analysis

To dissect the signaling pathways associated with PRMT expression across various cancers, patients were stratified into high- and low-expression groups based on the top and bottom 30% of each gene's expression levels, respectively. Differentially expressed genes between these cohorts were identified using the “limma” R package, applying a threshold of |log2 fold change| ≥ 1.0. Subsequently, Gene Set Enrichment Analysis (GSEA) was conducted via the “clusterProfiler” R package, referencing the Hallmark gene sets from the Molecular Signatures Database (MSigDB, v7.5.1) 34.

### Survival Analysis

The association between PRMT expression and patient prognosis was systematically evaluated using univariate Cox proportional hazards regression models across 33 TCGA cancer types. Four clinical endpoints—overall survival (OS), disease-specific survival (DSS), disease-free interval (DFI), and progression-free interval (PFI)—were included to provide a comprehensive assessment of their clinical relevance. Genes with a hazard ratio (HR) > 1 and *P* < 0.05 were defined as prognostic risk factors, whereas those with HR < 1 and *P* < 0.05 were identified as protective factors. Genes failing to meet the significance threshold (*P* ≥ 0.05) were deemed to have no significant impact on survival.

### Tumor Microenvironment (TME) Characterization

To evaluate the TME landscape, we utilized the ESTIMATE algorithm to calculate stromal, immune, and ESTIMATE scores, as well as tumor purity, across the pan-cancer cohort 35. The infiltration levels of various immune cell populations were quantified using the MCP-counter method 36. Furthermore, to dissect the TME-associated functional landscape, the IOBR package 37 was leveraged to assess several biological signatures, including immune activation, stromal remodeling, cell proliferation, and DNA damage repair (DDR). We then performed correlation analyses between PRMTs expression and these functional scores. Finally, we examined the expression of six classical immune checkpoints to further elucidate the immunomodulatory role of PRMTs.

### Cell Culture and RNA Interference

Human CRC cell lines (CACO2, HCT116, HCT8, HT29, LOVO, RKO, SW480, and SW620) and the normal colonic epithelial cell line NCM460 were obtained from the American Type Culture Collection. Cells were cultured in RPMI 1640 medium (Biological Industries, Israel) supplemented with 10% fetal bovine serum (Gibco, USA) and 100 U/mL penicillin-streptomycin (Invitrogen, USA) at 37°C in a humidified incubator with 5% CO_2_. For RNA interference, PRMT1-specific small interfering RNAs (siRNAs) were custom-synthesized by RiboBio (Guangzhou, China). The siRNA sequences were as follows: si#1, 5'-GCAACTCCATGTTTCATAA-3'; si#2, 5'-AGACGGTGTTCTACATGGA-3'. Transfections were performed using Lipofectamine 3000 (Invitrogen, USA) according to the manufacturer's protocol.

### RNA Extraction and Quantitative real-time PCR (qRT-PCR)

Total RNA was isolated from CRC cell lines using TRIzol reagent (Vazyme, Nanjing, China). cDNA was synthesized using the GoScript Reverse Transcription System (Promega, Madison, WI, USA) according to the manufacturer's protocol. qRT-PCR was performed with GoTaq qPCR Master Mix (Promega) on an ABI Prism 7500 Real-Time PCR System (Applied Biosystems, Foster City, CA, USA). Relative gene expression levels were calculated using the 2-^ΔΔCT^ method, with GAPDH serving as the internal control. The primer sequences used were as follows: PRMT1 forward, 5'-ACTTTGACTCCTACGCACACTTTG-3' and reverse, 5'-TCCAGCACCACCTTGTCCTTG-3'; GAPDH forward, 5'-AACGGATTTGGTCGTATTGG-3' and reverse, 5'-TTGATTTTGGAGGGATCTCG-3'.

### Western Blot Analysis

Total protein was extracted from cells using RIPA lysis buffer (supplemented with protease inhibitors). Equal amounts of protein were resolved by 10% SDS-PAGE and subsequently transferred onto PVDF membranes (Millipore, USA). After blocking with 5% non-fat milk for 1 h at room temperature, the membranes were probed overnight at 4 °C with primary antibodies against PRMT1 (1:1000, A1055, Abclonal, China) and GAPDH (1:2000, UM4002, Utibody, China). Following three washes with TBST, the membranes were incubated with appropriate HRP-conjugated secondary antibodies for 2 h at room temperature. Immunoreactive bands were visualized using an enhanced chemiluminescence kit.

### Immunohistochemical Analysis

IHC was performed following a previously established protocol 38. Briefly, 4-μm-thick CRC TMAs underwent dewaxing and hydration using xylene and a gradient of ethanol concentrations. Antigen retrieval involved microwave heating in sodium citrate buffer for 18 minutes. Endogenous peroxidase activity was blocked with 3% H_2_O_2_ for 10 minutes, and non-specific staining was reduced using 5% BSA. Anti-PRMT1 (1:500, A1055, Abclonal, China) was applied dropwise, followed by overnight incubation at 4 °C. A 20-minute incubation with anti-mouse/rabbit labeled polymer ensued. The exposed protein was labeled using a 0.01% DAB chromogenic solution, with nuclei counterstained using hematoxylin. Staining evaluation was independently performed by two pathologists blinded to clinicopathological information.

### CCK-8 Assay

Twenty-four hours post-transfection with siRNAs (si-NC, si-#1, and si-#2), cells were trypsinized and seeded into 96-well plates at a density of 3,000 cells/well. After an additional 24-hour incubation to allow for cell attachment, cell viability was evaluated using a CCK-8 assay kit (Dojindo, Kumamoto, Japan) according to the manufacturer's instructions. Briefly, CCK-8 reagent was diluted 1:10 with fresh culture medium and added to each well. After incubation at 37 °C for 2 hours, the absorbance at 450 nm was measured using a microplate reader. To monitor cell growth over time, this procedure was repeated at 24, 48, 72, and 96 hours post-seeding.

### Clonogenic Formation Assay

Following siRNA transfection, cells were harvested, resuspended, and seeded at a density of 600 cells/well in 12-well plates. The culture medium was refreshed every 2-3 days. After one week, the cell colonies were fixed with 4% paraformaldehyde for 20 minutes, and subsequently stained with 0.1% crystal violet solution for 30 minutes. After washing to remove excess dye, the plates were air-dried and photographed. The number of colonies was then quantified for further analysis.

### EdU Proliferation Assay

Cell proliferation was also assessed using the BeyoClick™ EdU 594 Cell Proliferation Detection Kit (Beyotime, Shanghai, China). Briefly, 24 hours post-transfection, cells were pulse-labeled with 10 μM EdU for 2 hours. Following incubation, cells were fixed with 4% paraformaldehyde for 15 minutes and permeabilized with 0.1% Triton X-100 for 10 minutes. The click reaction was then performed in the dark for 30 minutes according to the manufacturer's instructions. Nuclei were counterstained with Hoechst 33342. Fluorescence images were captured using a fluorescence microscope to quantify the percentage of EdU-positive cells.

### Statistical Analysis

All statistical analyses were conducted using the R (version 4.3.1, https://www.r-project.org/) and GraphPad Prism 9. For normally distributed variables, differences between two groups were assessed using Student's t-test, and multiple groups were compared via one-way ANOVA. Non-normally distributed variables were analyzed using the Wilcoxon test for two groups and the Kruskal-Wallis test for multiple groups. Pearson correlation analysis was utilized to calculate correlation coefficients. Heatmaps were generated using the “ComplexHeatmap” package. Results were reported as Mean ± SD. All statistical tests were two-tailed, and a *P*-value < 0.05 was considered statistically significant (*P < 0.05, **P < 0.01, ***P < 0.001, ****P < 0.0001).

## Results

### Expression Profiling of PRMTs Across Pan-Cancer

**Figure 1:**
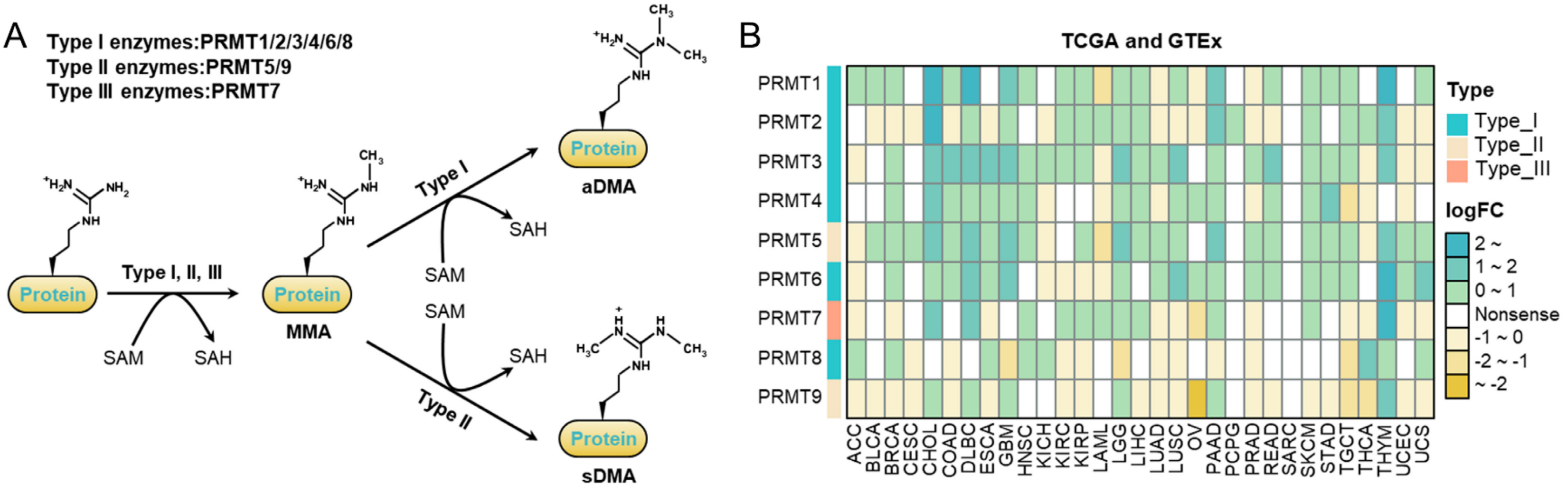
** Characterization and expression analysis of PRMTs across human cancers.** (A) Schematic representation of protein arginine methylation, illustrating the classification and catalytic mechanisms of the three PRMT types on arginine residues. (B) Differential mRNA expression of PRMTs across TCGA and GTEx cohorts. Fold changes and *P* values were determined by comparing tumor tissues (TCGA) with their corresponding normal counterparts (GTEx and TCGA). Heatmap colors represent the magnitude of fold change; “Nonsense” denotes *P* value > 0.05.

To provide a foundation for our study, we first summarized the classification and catalytic mechanisms of the PRMT family (Figure 1A). Based on their specific catalytic products, PRMTs are categorized into three types: Type I (PRMT1-4, 6, and 8), which generate monomethylarginine (MMA) and asymmetric dimethylarginine (aDMA); Type II (PRMT5 and PRMT9), which produce MMA and symmetric dimethylarginine (sDMA); and Type III (PRMT7), which exclusively catalyzes the formation of MMA (Figure 1A).

Leveraging TCGA and GTEx datasets, pan-cancer transcriptomic analysis revealed widespread dysregulation of PRMTs across various malignancies (Figure 1B, S1A-B). Most members, particularly PRMT1 and PRMT3-7, showed significant and consistent overexpression across diverse tumor types, with prominent upregulation observed in gastrointestinal and thoracic cancers (Figure 1B, S1A-B). In contrast, PRMT2 and PRMT9 displayed a distinct downregulation pattern in specific cancers, such as ovarian serous cystadenocarcinoma (OV), suggesting potential functional divergence within the PRMT family (Figure 1B, S1A-B).

### Genetic Alterations and Epigenetic Regulation of PRMTs in Pan-Cancer

**Figure 2:**
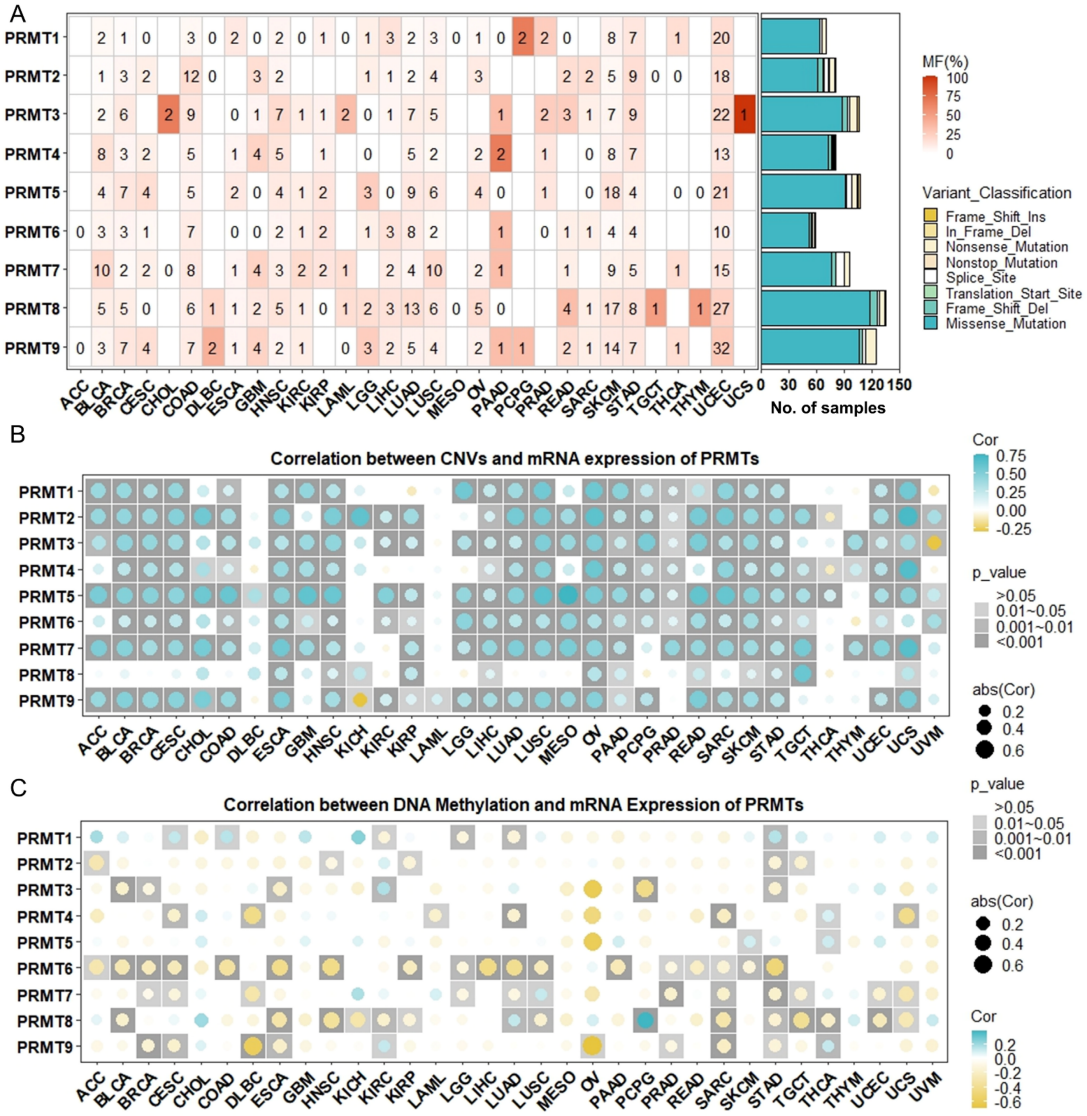
** Genetic and epigenetic landscape of PRMTs across pan-cancer.** (A) Mutational profile of PRMTs across cancer types. The left heatmap displays the number of mutated samples for each PRMT gene; '0' denotes no mutations within coding regions, and blank cells indicate no detected mutations. The right stacked bar chart summarizes the distribution of specific variant types across the PRMT family. (B-C) Correlation of PRMT mRNA expression with (B) CNVs and (C) DNA methylation. Circle size and color represent the absolute correlation coefficient and the correlation value, respectively, while the background color of each cell indicates statistical significance (P value).

To characterize the genetic alterations of PRMTs, we analyzed single nucleotide variations (SNVs) across pan-cancer cohorts. The highest mutation frequency was observed in UCEC, particularly affecting PRMT3, 8, and 9, with missense mutations representing the predominant variant type (Figure 2A). In contrast, PRMTs displayed low mutation frequencies in most other tumor types (Figure 2A). Lollipop plots further revealed that these mutations were stochastically distributed along the protein sequences, lacking significant enrichment within specific functional domains (Figure S2A). Taken together, the low overall mutation frequency and sporadic distribution suggest that PRMT-driven oncogenesis is likely orchestrated by transcriptional or epigenetic dysregulation rather than recurrent driver mutations.

Aberrant mRNA expression in cancer is frequently governed by a synergy of genomic instability and epigenetic modifications, with CNVs and DNA methylation acting as primary regulatory determinants. To further investigate the mechanisms underlying PRMT dysregulation, we first assessed the frequency of CNV alterations across various malignancies. Our results revealed that PRMT genes exhibited a relatively high frequency of CNV alterations (≥5% of samples) in most cancer types (Figure S2B). Subsequent correlation analyses showed a significant positive association between CNV levels and mRNA expression for these genes in the majority of tumors, with notable exceptions in DLBC, KICH, LAML, and THCA (Figure 2B). These findings suggest that CNVs play a major role in the aberrant expression of PRMTs and may contribute significantly to cancer progression.

Beyond structural genomic alterations, DNA promoter methylation is a key epigenetic regulator of gene transcription. Evaluation of the DNA methylation landscapes of PRMTs showed that significant divergence between tumor and normal tissues was largely absent across the family, with PRMT8 being the notable exception (Figure S2C). In line with this, correlation analysis confirmed that promoter methylation levels rarely tracked with mRNA expression in most cancers (Figure 2C). These results indicate that DNA methylation is likely not the predominant driver of PRMT dysregulation.

### Prognostic Implications of PRMTs in Pan-Cancer

**Figure 3:**
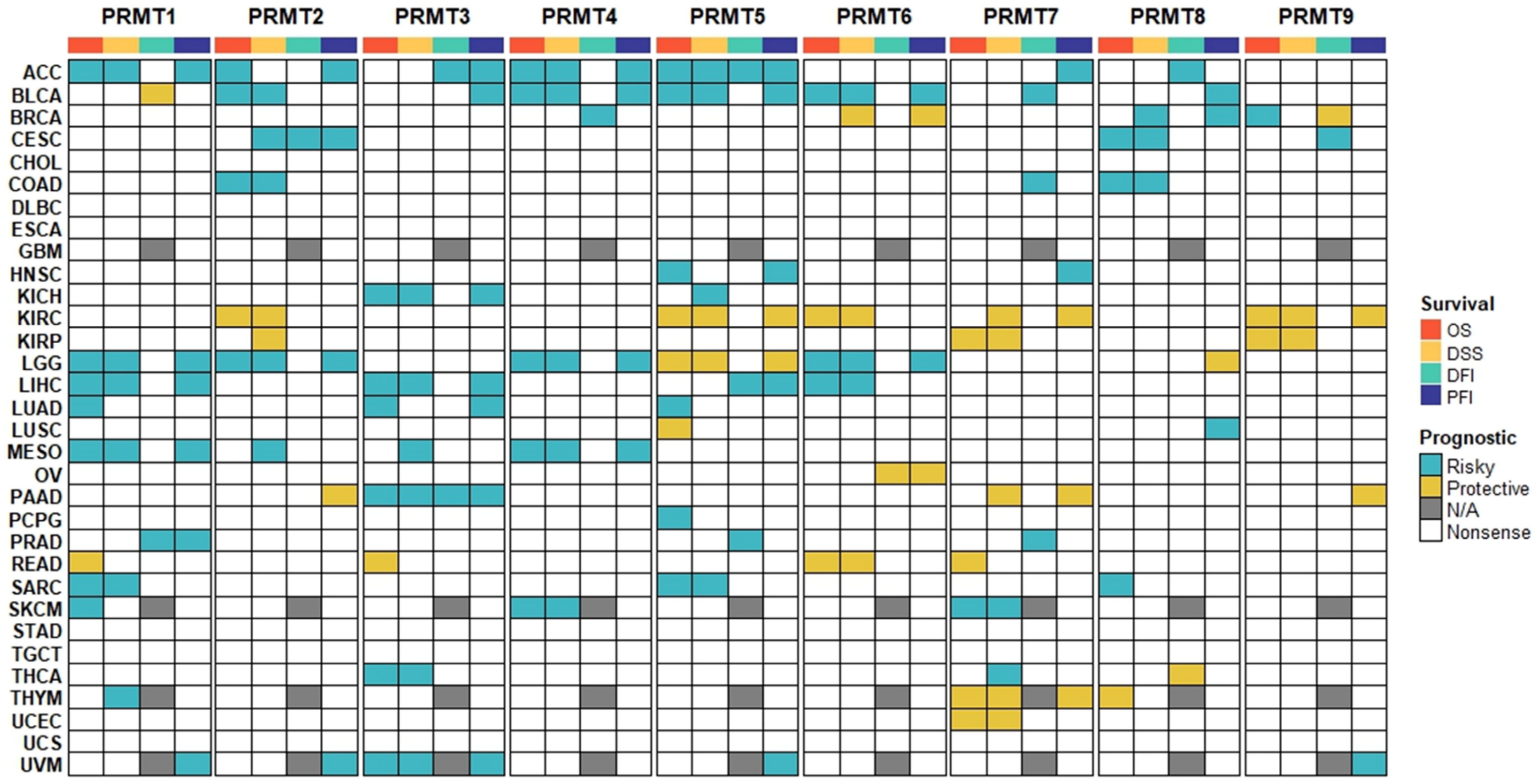
** Pan-cancer Prognostic Significance of PRMTs.** Heatmap illustrating the associations between PRMT expression and clinical outcomes—including OS, DSS, DFI, and PFI—across 32 TCGA cancer types, as determined by univariate Cox regression analysis. Turquoise denotes a risk factor (unfavorable prognosis), whereas yellow denotes a protective factor (favorable prognosis). White indicates no statistical significance, and “N/A” represents unavailable data.

To further evaluate the clinical significance of PRMTs, we examined the correlation between their expression profiles and patient prognosis, focusing on OS, DSS, DFI, and PFI. Univariate Cox regression analysis of the TCGA pan-cancer dataset revealed that while the prognostic values of PRMTs were highly heterogeneous across diverse malignancies, a discernible, albeit modest, consistency in prognostic orientation was observed within specific tumor types (Figure 3). Specifically, instead of acting as isolated factors, PRMT family members often exhibited a unified trend in their impact on patient survival within the same cancer context. For instance, in adrenocortical carcinoma (ACC), elevated expression of PRMT1-5 tended to associate with unfavorable clinical outcomes, particularly shortened OS and PFI (Figure 3). Conversely, in kidney renal clear cell carcinoma (KIRC), reduced expression of PRMT2, 5, 6, 7, and 9 showed a general correlation with poorer survival, suggesting a potentially protective role for these genes in this specific malignancy (Figure 3).

Furthermore, we investigated the prognostic impact of individual PRMTs across the pan-cancer spectrum. While PRMT1-4 frequently leaned toward being poor prognostic indicators in a wide range of cancers, PRMT5-7 exhibited dual roles, with their prognostic effects varying significantly depending on the cancer type (Figure 3). Collectively, these findings highlight the complex and context-dependent prognostic roles of PRMTs across the pan-cancer landscape, underscoring the need for further functional studies to elucidate their divergent roles in tumor biology.

### Correlation of PRMTs with Genomic Heterogeneity and Stemness Indices

The prognostic impact of PRMTs prompted us to explore their links to genomic instability—a driver of tumor evolution and therapeutic resistance. Systemic analysis revealed that PRMT1, 3, 4, and 5 expression positively correlated with HRD, LOH, and ploidy across various cancers (e.g., KICH, LIHC, LUAD, LUSC), suggesting their role in exacerbating chromosomal aberrations (Figure S3). Regarding mutational landscape, the PRMT family exhibited a pro-mutational profile. Specifically, PRMT1, 4, and 5 levels were linked to increased TMB and neoantigen counts in several cancers (ACC, HNSC, LUAD, etc.) (Figure S3). Notably, PRMT1/8 (in KICH) and PRMT4/7 (in UVM) showed significant positive correlations with MSI status, potentially implying their involvement in the dysregulation of DNA mismatch repair pathways (Figure S3). Furthermore, positive correlations between PRMT1, 3, 5 and MATH scores indicated higher intratumoral heterogeneity and subclonal complexity (Figure S3). Intriguingly, PRMT expression positively correlated with tumor purity in most cases (Figure S3). This confirms that PRMTs are primarily tumor-intrinsic and ensures that the observed genomic heterogeneity is not confounded by stromal cell dilution.

As tumor stem-like cells are pivotal to heterogeneity and therapy resistance, we next assessed the association between PRMT expression and tumor dedifferentiation using six stemness indices (Figure S4). While PRMT1, 3, 4, and 5 positively correlated with stemness across multiple cancers, PRMT2 exhibited a predominantly negative correlation, highlighting functional divergence within the family (Figure S4). Interestingly, the correlations were more pronounced with RNAss (transcriptome-based) than with epigenetic indices (e.g., DNAss, DMPss) (Figure S4), suggesting that PRMTs influence stemness mainly at the transcriptional and post-transcriptional levels. These findings imply that PRMTs may drive the dedifferentiation landscape by modulating the methylation of key transcription or splicing factors.

### Deciphering the Relationship between PRMTs and Tumor Microenvironment Across Pan-Cancer

**Figure 4:**
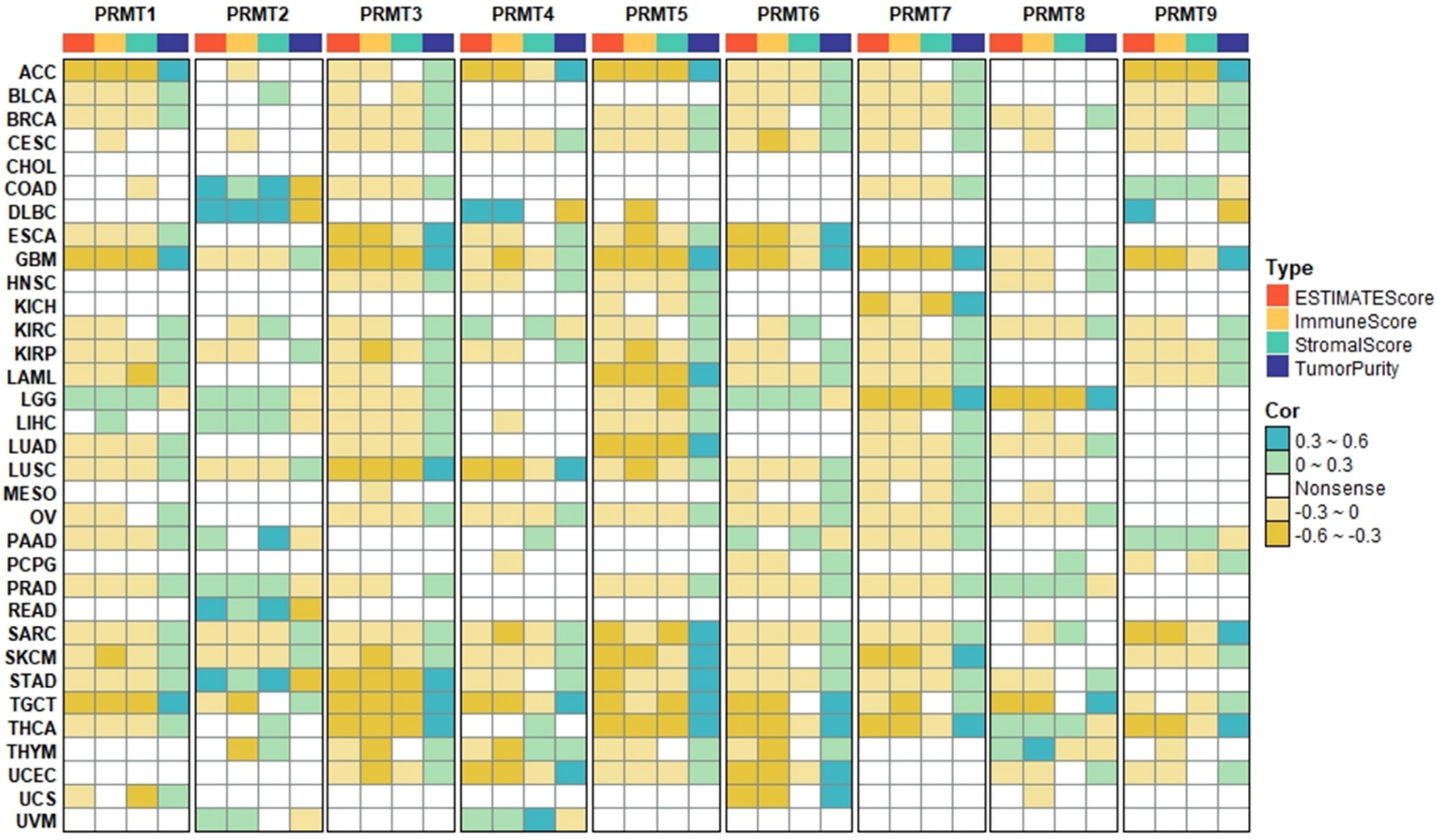
** Associations of PRMTs with TME scores across pan-cancer.** Heatmap showing the correlation of PRMT expression with immune, stromal, and ESTIMATE scores, as well as tumor purity. Turquoise and yellow represent positive and negative correlations, respectively, while white indicates a lack of statistical significance.

Cancer progression is governed by “immunoediting,” where the immune system either eliminates malignant cells or fosters a tumor-permissive microenvironment 39. Utilizing the ESTIMATE algorithm, we identified that most PRMTs (PRMT1, 3-9) are negatively associated with immune/stromal scores and positively with tumor purity, especially in GBM, STAD, TGCT, and THCA (Figures 4). However, PRMT2 emerged as an outlier, showing positive correlations with immune/stromal scores in COAD, DLBC, and READ (Figure 4).

Subsequent lineage-specific quantification via MCP-counter revealed that while PRMT1, 3, and 5 inversely correlated with anti-tumor effectors (CD8⁺ T, cytotoxic lymphocytes, and NK cells), PRMT2 expression positively associated with these cells as well as stromal and myeloid components (Figure S5). Considering that PRMT2 is frequently downregulated in tumors (Figures 1B, S1A-B), its loss likely drives a transition toward an “immune-cold” state. Notably, MCP-counter provided a more nuanced view for PRMT4, 6, and 9 than the aggregate ESTIMATE scores, uncovering positive associations with endothelial cells and fibroblasts despite negative global trends (Figures 4, S5).

To further dissect the molecular underpinnings of PRMT-mediated TME remodeling, we evaluated the correlations between PRMT expression and several curated functional signatures, encompassing immune activation, stromal remodeling, and tumor-intrinsic proliferative processes. The results demonstrated that PRMT1, 3, 4, 5, 6, and 7 were robustly associated with cell-cycle progression and DDR pathways, while exhibiting modest inverse correlations with immune activation and stromal remodeling signatures (Figure S6). This underscores their primary role in maintaining genomic integrity and driving oncogenic proliferation. Conversely, PRMT2 displayed a unique functional profile, correlating positively with immune activation and stromal remodeling markers across a subset of malignancies (Figure S6). Consistently, further analysis revealed that PRMT2 was also positively associated with the expression of multiple immune checkpoints, further distinguishing it from other family members (Figure S7).

### Correlation Between PRMTs and Hallmark Pathways in Pan-Cancer

**Figure 5:**
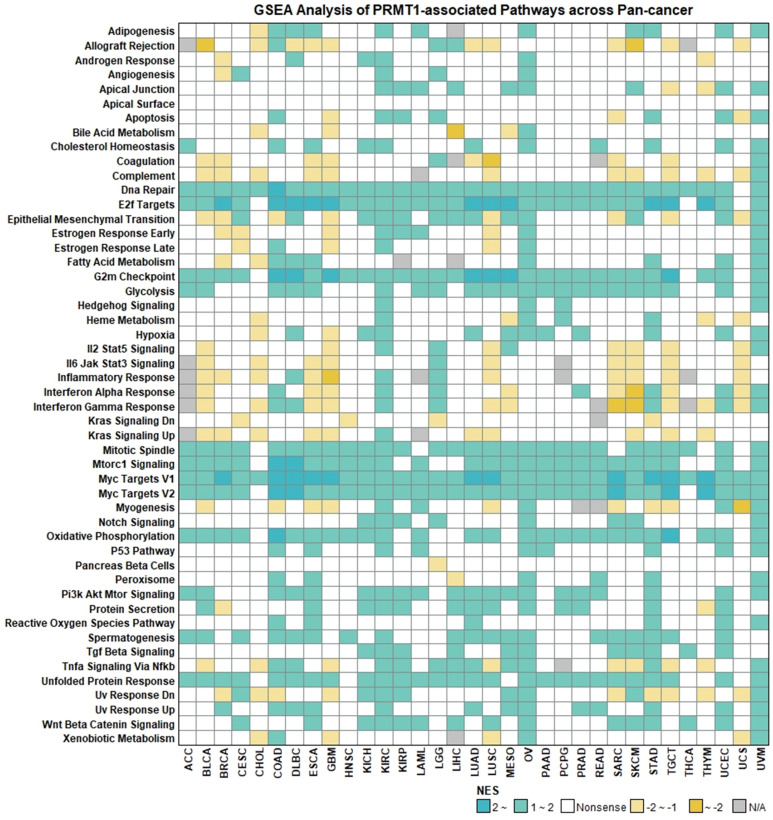
** Associations between PRMT1 and Hallmark Pathways across Pan-Cancer.** Colors represent the normalized enrichment score (NES) for each pathway, and “Nonsense” indicates P > 0.05.

To broaden our understanding of the biological roles of PRMTs, we performed GSEA to identify enriched Hallmark pathways across pan-cancer datasets. This profiling aimed to consolidate our previous findings regarding the cell cycle and DDR while unearthing novel signaling axes through which PRMTs might modulate tumor progression and the immune landscape. Recapitulating our earlier observations, we found that RMT1, 3, 5, 6, 7, and 9 predominantly activated oncogenic and proliferative pathways, but were negatively coupled with immune-related signatures (Figure S8). Using PRMT1 as a representative model, high expression was significantly enriched in pathways governing cell-cycle progression and metabolic fitness, including DNA repair, E2F targets, G2M checkpoint, mTORC1 signaling, MYC targets, and oxidative phosphorylation (Figure 5). Furthermore, PRMT1 displayed positive associations with pro-survival cascades such as PI3K/AKT/mTOR and Wnt/β-catenin (Figure 5). In contrast, PRMT1 expression was inversely correlated with multiple immuno-stimulatory and pro-inflammatory signatures, including allograft rejection, IL2/STAT5, interferon-α/γ responses, and the IL6/JAK/STAT3 axis (Figure 5). Notably, PRMT2 exhibited a functional antithesis to its counterparts, positively correlating with immune-regulatory and inflammatory pathways (Figure S8), reinforcing its unique role in the TME.

To determine whether the oncogenic correlations of PRMTs translate into functional requirements for survival, we assessed their genetic dependency using DepMap CRISPR screening data. While a score of 0 indicates non-essentiality, a score ≤ -1 represents core essentiality 40, 41. We found that PRMT1 and PRMT5 are critical for cell growth across most cancer lines (Figure S9), reflecting their potent oncogenic roles. Interestingly, despite their association with proliferative programs in GSEA, PRMT3, 6, 7, and 9 yielded dependency scores near zero (Figures S8, S9). This suggests that these PRMTs may be functionally redundant or serve as context-specific modulators rather than indispensable survival factors.

### The Expression Landscape of PRMTs in CRC

**Figure 6:**
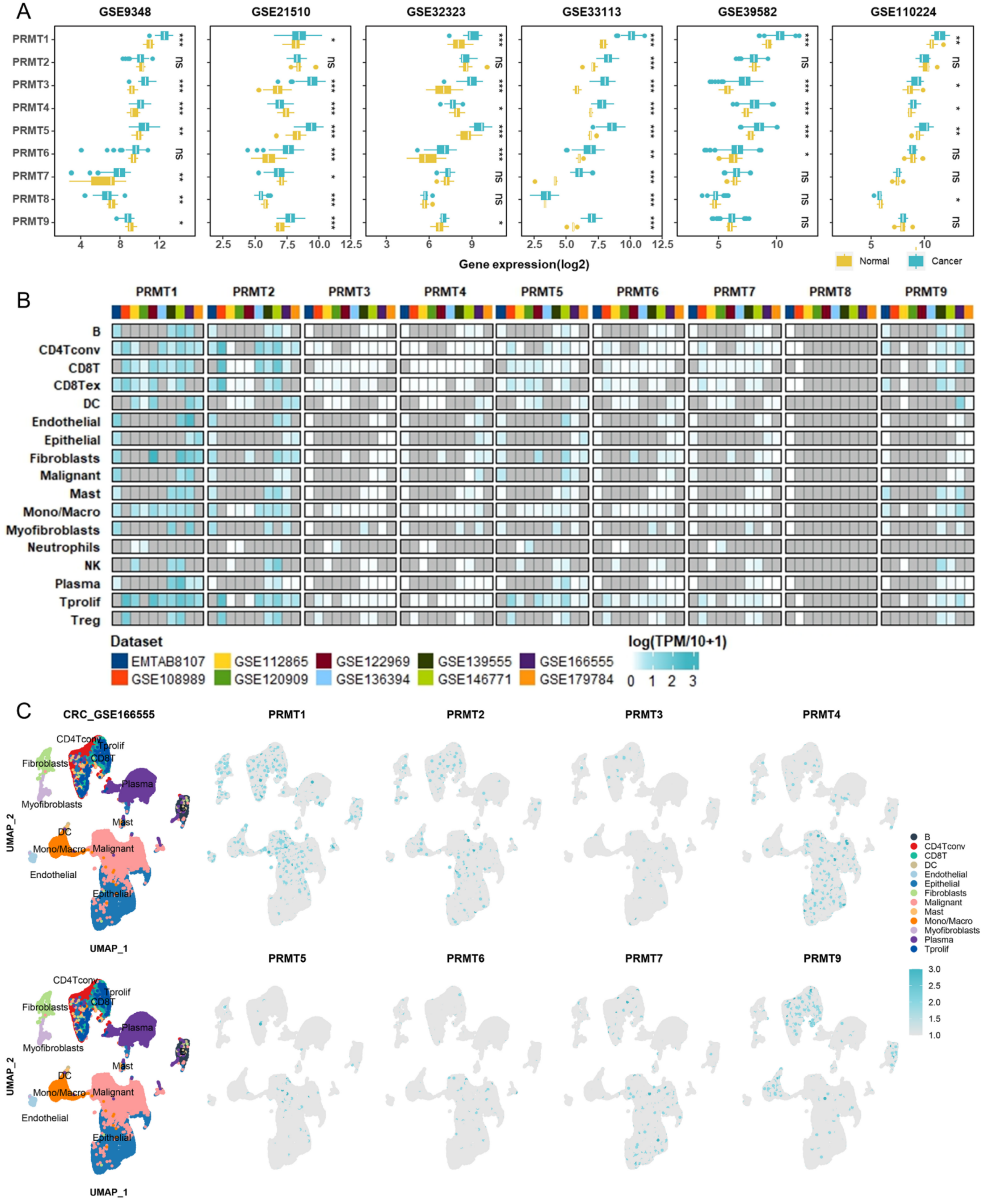
** Validation of PRMT expression in CRC using bulk and single-cell transcriptomics.** (A) Box plots showing the mRNA expression levels of PRMT family members across multiple independent CRC GEO cohorts. (B) Overview of PRMT expression across diverse cell populations in CRC scRNA-seq data. The color gradient represents the relative expression level, while gray indicates missing values or data not detected. (C) Scatter plots showing cell-type clustering and the expression of PRMT members in the GSE166555 dataset. Note that PRMT8 was excluded due to its absence in this dataset.

To validate our pan-cancer findings within a specific clinical context, we focused our investigation on CRC—a malignancy where PRMT-mediated epigenetic dysregulation is a recognized driver of progression. We first validated the expression profiles of PRMTs in CRC using multiple independent cohorts from the GEO database. Analysis of six GEO datasets revealed that PRMT1, 3, and 5 were consistently upregulated in tumor tissues (Figure 6A). In contrast, PRMT4 showed upregulation in four datasets but downregulation in two, while other PRMT members exhibited differential expression only in a subset of the cohorts (Figure 6A). Furthermore, to investigate the potential involvement of PRMTs in therapeutic response, we extended our analysis to six GEO datasets from patients receiving fluoropyrimidine-based chemotherapy, as well as three additional datasets involving bevacizumab treatment. Notably, significant differences in expression relative to treatment outcomes were observed only for PRMT3, 4, and 5 in a few specific datasets (Figures S10A-S10I), suggesting that PRMT expression levels may not be robustly associated with therapeutic efficacy. To address the cellular heterogeneity inherent in bulk transcriptomic data, we further investigated the single-cell expression patterns of PRMTs in CRC. Analysis of scRNA-seq data revealed that PRMT1 and PRMT2 were broadly distributed across immune, stromal, and malignant cell populations (Figures 6B, 6C). Collectively, these results indicate that although specific PRMTs (particularly PRMT1, 3, and 5) are consistently overexpressed in CRC, their transcript levels are not strong predictors of treatment response, and their widespread distribution suggests complex, multifaceted roles within the tumor microenvironment.

### PRMT1 Is Upregulated in CRC and Promotes CRC Cell Growth in Real-World Validation

**Figure 7:**
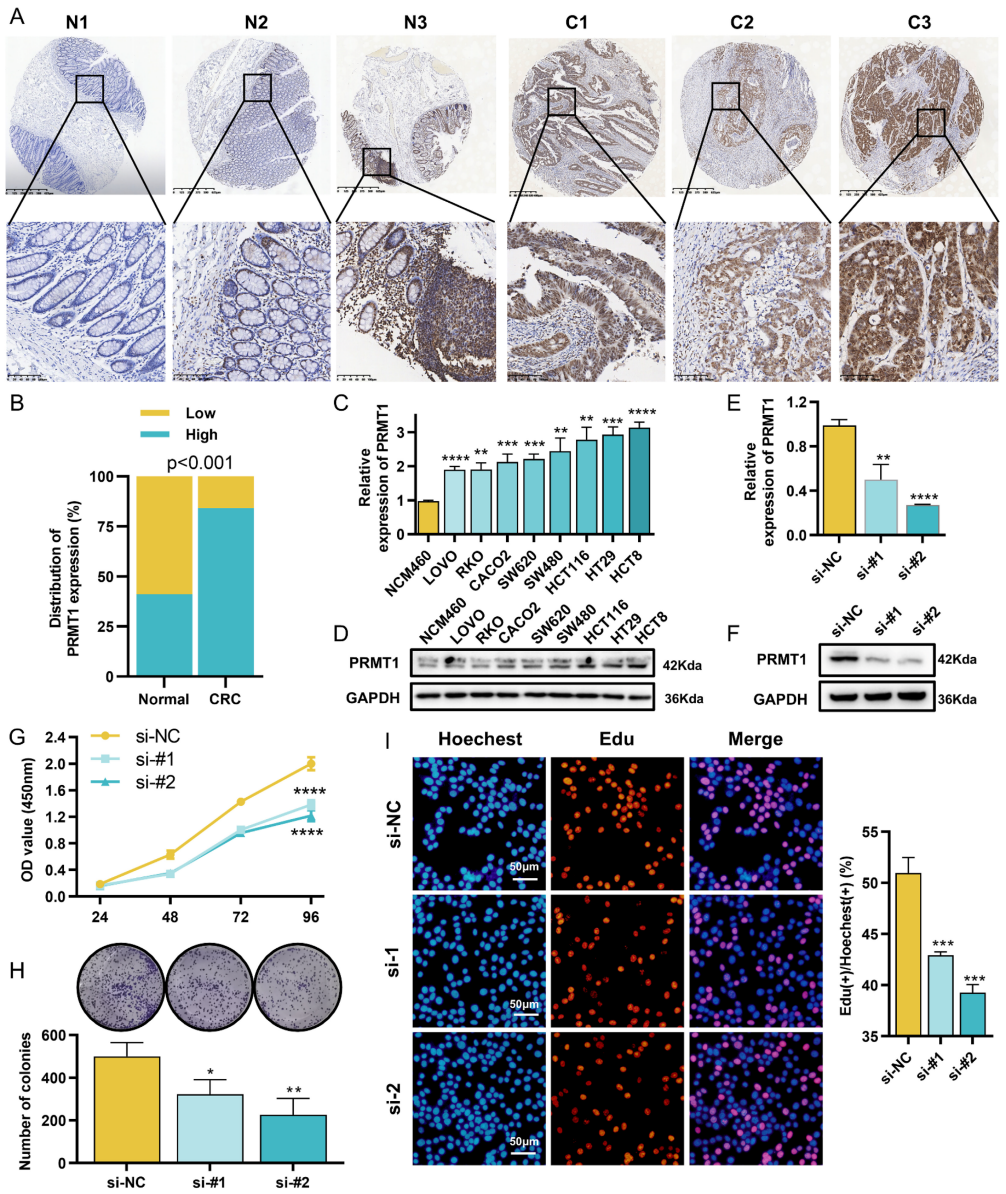
** PRMT1 is overexpressed in CRC and required for tumor cell growth.** (A) Representative IHC staining of PRMT1 in CRC tissues (C1-C3) and adjacent normal mucosa (N1-N3). (B) Quantification of PRMT1 expression in the clinical cohort, shown as positivity rates. (C, D) Basal expression levels of PRMT1 in CRC cell lines detected by qRT-PCR (C) and Western blotting (D). (E, F) Validation of PRMT1 knockdown efficiency in HCT8 cells at the mRNA level (E) and protein level (F). (G-I) Effects of PRMT1 knockdown on HCT8 cell proliferation, assessed by CCK-8 assay (G), colony formation assay (H), and EdU proliferation assay (I).

Given its elevated expression and functional dependency in CRC cells (Figures 1B, 6A-6C, S1A, S1B, S9), PRMT1 was prioritized for validation in a real-world clinical cohort. We first characterized the expression and spatial distribution of PRMT1 in paraffin-embedded tissues using IHC. In the normal mucosa, PRMT1 exhibited variable staining intensity across epithelial, stromal, and immune cells (Figure 7A). In contrast, PRMT1 expression was markedly higher in tumor cells compared to the relatively weak staining observed in the tumor stroma and infiltrating immune cells (Figure 7A). Regarding its subcellular localization, PRMT1 was predominantly restricted to the nucleus in both normal and malignant tissues (Figure 7A). Quantitative analysis revealed that the PRMT1 positivity rate was significantly higher in tumor tissues than in normal controls (Figure 7B). Furthermore, clinicopathological analysis demonstrated that high PRMT1 expression was significantly associated with larger tumor size, advanced T stage, and lymph node metastasis (Table 1). Consistent with these clinical findings, PRMT1 was found to be upregulated in CRC cell lines at both the mRNA and protein levels (Figures 7C, 7D).

Aligning with our pan-cancer observations linking PRMT1 to cell growth, we performed further validation using GEO datasets. GSVA and GSEA identified a positive correlation between PRMT1 expression and pathways related to the cell cycle and DDR (Figures S11A, S11B). To experimentally investigate the functional role of PRMT1, we performed siRNA-mediated knockdown in HCT8 cells, achieving substantial depletion of PRMT1 (Figures 7E, 7F). Subsequent functional assays—including CCK-8 (Figure 7G), colony formation (Figure 7H), and EdU proliferation assay (Figure 7I)—demonstrated that PRMT1 silencing significantly impaired HCT8 cell proliferation. Collectively, these findings establish PRMT1 as a clinically relevant oncogene in CRC that correlates with disease progression and is essential for maintaining the proliferative capacity of tumor cells.

## Discussion

PRMTs play crucial roles in cancer biology, regulating processes like gene expression, signal transduction, and DNA repair 9, 42, 43. In this study, we systematically characterized the pan-cancer expression landscape of PRMTs using TCGA and GTEx datasets. Our findings reveal widespread dysregulation of PRMTs across various malignancies. Specifically, PRMT1, 3, 4, 5, 6, and 7 were predominantly upregulated, while PRMT2 and PRMT9 were consistently downregulated, suggesting divergent roles for these proteins in tumorigenesis. Mechanistically, we identified CNV-driven genomic instability as the primary driver of PRMT dysregulation, showing a strong positive correlation with mRNA levels. In contrast, DNA methylation exhibited a limited regulatory impact. Furthermore, the prognostic significance of PRMTs appeared highly context-dependent: high expression of PRMT1, 3, and 4 consistently predicted poor survival, supporting their roles as oncogenic drivers, whereas PRMT2, 5, 6, 7, and 9 showed tumor-specific prognostic effects. These findings suggest that the coordinated but distinct dysregulation of PRMT family members contributes to tumor progression in a context-specific manner.

The maintenance of genomic integrity is orchestrated by a sophisticated network of surveillance systems, including DNA damage checkpoints and repair machineries 44. PRMT family members emerge as pivotal regulators in this context, directly impacting genomic stability through the modulation of DNA repair pathways 5. Specifically, PRMT1, 4, and 5 facilitate homologous recombination by arginine methylating key repair factors, including MRE11, hnRNPUL1, and BRCA1 45-47. Moreover, PRMT1 and PRMT5 have been shown to influence non-homologous end joining through the regulation of 53BP1 45, 48, while PRMT1, 5, and 6 contribute to base excision repair, highlighting their multifaceted contributions to genomic preservation 49, 50. Consistent with these roles, our study reveals a strong correlation between the expression of PRMT1, 3, 4, and 5 and clinical markers of genomic damage (HRD, LOH, ploidy, and MSI). Enrichment analyses further underscore the involvement of PRMTs in cell proliferation and DDR pathways. Collectively, these results suggest that PRMT dysregulation fuels the accumulation of genomic heterogeneity by disrupting DNA repair, thereby propelling malignant transformation.

CSCs are fundamental orchestrators of tumor progression and treatment failure, owing to their robust self-renewal and plasticity 51. Evidence indicates that PRMTs orchestrate CSC biological behavior through the arginine methylation of key transcription factors (e.g., OCT4, SOX2, and MYC) and the modulation of canonical oncogenic pathways, such as WNT and RAS signaling 5. In the present study, we observed that PRMT expression levels significantly correlated with the transcriptomic stemness index (RNAss), while showing relatively weak associations with epigenetic stemness indices (e.g., DNAss and DMPss). This disparity suggests that PRMTs may modulate tumor plasticity primarily through post-transcriptional mechanisms, potentially by methylating key transcription factors or splicing regulators. Moreover, functional enrichment analysis validated the pervasive positive associations between PRMTs and key stemness-regulatory networks (e.g., Wnt, Hedgehog, and TGF-β). Taken together, these data highlight PRMTs as central regulators of tumor heterogeneity and malignant progression, providing a novel framework for precision-targeted interventions against the CSC niche.

The TME, an intricate ecosystem of immune cells, stroma, and extracellular matrix, maintains a reciprocal relationship with the cellular epigenome 52, 53. While TME-derived physicochemical cues trigger epigenetic reprogramming, the subsequent epigenetic shifts in tumor and stromal cells feed back to reshape the TME, fueling malignancy and immune evasion 53. As pivotal epigenetic modifiers, PRMTs are deeply involved in anti-tumor immune responses by modulating innate immune signaling pathways and the expression of key molecules such as PD-L1 and MHC-I 5. Notably, targeting PRMT1 or PRMT5 has been shown to alleviate immunosuppression and sensitize tumors to PD-1/PD-L1 blockade 54, 55. Here, we characterize the functional divergence of the PRMT family in TME remodeling. Upregulation of PRMT1, 3, and 5, alongside PRMT2 downregulation, characterizes “immune-excluded” or “cold” tumor profiles, indicating a synergistic contribution to immune surveillance escape. Conversely, PRMT4, 6, and 9 may indirectly modulate the TME through stromal expansion and angiogenesis, as evidenced by their positive correlation with fibroblast and endothelial infiltration despite negative associations with immune scores. The pronounced inter-cancer heterogeneity of PRMT-related immune landscapes underscores the need for precision oncology. While these findings highlight the PRMT-TME nexus, further *in vivo* and *in vitro* mechanistic studies are required. Overall, decoding the crosstalk between PRMTs and the TME provides a theoretical foundation for understanding immunoediting and optimizing combinatorial immunotherapy.

We selected CRC, a globally prevalent and highly heterogeneous malignancy, as a representative model to validate our pan-cancer findings 20, 56. While PRMT1 and PRMT5 are known oncogenic drivers in CRC 14, 57, the collective impact of the PRMT family on CRC progression remains to be systematically elucidated. Our analysis of six independent GEO cohorts confirmed the consistent upregulation of PRMT1, 3, and 5, mirroring our pan-cancer results. Notably, in our clinical cohort, PRMT1 overexpression was positively associated with tumor size, T-stage, and nodal metastasis, highlighting its clinical relevance. This was further supported by functional evidence: both CRISPR-Cas9 screen data and *in vitro* silencing of PRMT1 demonstrated a significant suppression of CRC cell growth. Mechanistically, PRMT1 was found to be tightly linked to cell proliferation and DDR signaling, implying that PRMT1 supports CRC malignancy by maintaining genomic integrity and cell cycle progression. Collectively, these multi-dimensional insights—bridging bioinformatics, clinical data, and functional validation—identify PRMT1 as a central regulator of CRC growth and a promising target for precision therapy.

Despite the significant insights offered by this pan-cancer analysis of the PRMT family, certain limitations remain. A primary constraint is the reliance on public repositories (TCGA, GTEx, and GEO), where inter-cohort heterogeneity in sequencing methodologies and sample sizes may impact the robustness of our conclusions. To enhance the universality of these findings, future integration of multi-center clinical data is required. Furthermore, the scope of our functional validation was largely restricted to PRMT1 in the context of colorectal cancer. While these findings are promising, the mechanistic underpinnings of PRMT1 in other oncogenic contexts, as well as the roles of other PRMT family members, remain to be systematically elucidated. Prospective studies should encompass the full spectrum of the PRMT family across diverse cancer models. Lastly, the observed associations between PRMTs and the immune microenvironment necessitate further mechanistic confirmation. Investigating the translational potential of PRMTs as targets for combinatorial immunotherapy will be essential for advancing precision oncology and optimizing therapeutic strategies.

## Conclusion

In summary, our multi-omic analysis demonstrates that the PRMT family is extensively dysregulated across cancers, driving genomic instability, cancer stemness, and immune evasion. Specifically, our validation of PRMT1 in CRC underscores its clinical significance and its critical role in tumor progression. By integrating pan-cancer data with experimental validation, this study establishes the PRMT family as a key therapeutic target and provides a foundation for personalized cancer treatment strategies.

## Supplementary Material

Supplementary figures.

## Figures and Tables

**Table 1:** Correlations between PRMT1 expression and clinicopathologic features in 120 CRC patients.

Clinicopathological feature		Expression of PRMT1		*P* value
	Total (n=120)	Low (n=19)	High (n=101)	
Gender				
Male	83	12	71	0.584
Femal	37	7	30	
Age				
< 60	67	10	57	0.803
≥ 60	53	9	44	
Tumor size				
≤ 5 cm	79	17	62	0.021
> 5 cm	41	2	39	
Tumor location				
Colon	70	9	61	0.316
Rectum	50	10	40	
Differentiation				
Well and Moderate	107	18	89	1
Poor	13	1	12	
T stage				
T1-2	15	11	4	<0.001
T3-4	105	8	97	
Lymph node metastasis				
No	73	16	57	0.027
Yes	47	3	44	
Distant metastasis				
No	115	19	96	1
Yes	5	0	5	

## Data Availability

The datasets supporting the findings of this study are available from the corresponding author upon reasonable request.

## Abbreviations

ACC: adrenocortical carcinoma
aDMA: asymmetric dimethylarginine
BLCA: bladder urothelial carcinoma
BRCA: breast invasive carcinoma
CHOL: cholangiocarcinoma
CNV: copy number variation
COAD: colon adenocarcinoma
CRC: colorectal cancer
DDR: DNA damage repair
DFI: disease-free interval
DLBCL: diffuse large B-cell lymphoma
DSS: disease-specific survival
ESCA: esophageal carcinoma
GBM: glioblastoma multiforme
GEO: gene expression omnibus
GSEA: gene set enrichment analysis
GTEx: genotype-tissue expression
HNSC: head and neck squamous cell carcinoma
HR: hazard ratio
HRD: homologous recombination deficiency
IF: immunofluorescence
IHC: immunohistochemistry
KICH: kidney chromophobe
KIRC: kidney renal clear cell carcinoma
LAML: acute myeloid leukemia
LGG: lower grade glioma
LIHC: liver hepatocellular carcinoma
LOH: loss of heterozygosity
LUAD: lung adenocarcinoma
LUSC: lung squamous cell carcinoma
MATH: mutant-allele tumor heterogeneity
MMA: monomethylarginine
MSI: microsatellite instability
MSigDB: molecular signatures database
NES: normalized enrichment score
OS: overall survival
OV: ovarian serous cystadenocarcinoma
PFI: progression-free interval
PRMT: protein arginine methyltransferase
qRT-PCR: quantitative real-time polymerase chain reaction
SARC: sarcoma
scRNA-seq: single-cell RNA sequencing
sDMA: symmetric dimethylarginine
siRNAs: small interfering RNAs
SNVs: single nucleotide variants
STAD: stomach adenocarcinoma
TCGA: the cancer genome atlas
TGCT: testicular germ cell tumors
THCA: thyroid carcinoma
THYM: thymoma
TMA: tissue microarray
TMB: tumor mutational burden
TME: tumor microenvironment
UCEC: uterine corpus endometrial carcinoma
UCSC: the University of California, Santa Cruz
