# Supplementary material for: Multi-Omics and Clinical Data Analyses of Protein Arginine Methyltransferases in Pan-Cancer and Colorectal Cancer
Source: Int J Med Sci. 2026 Jul 13;23(8):2621–36. doi: 10.7150/ijms.129345 (PMC13411392; doi:10.7150/ijms.129345)
Supplement: Supplementary file 1 — Supplementary figures. [file ijmsv23p2621s1.pdf]

Supplemental Figure legends

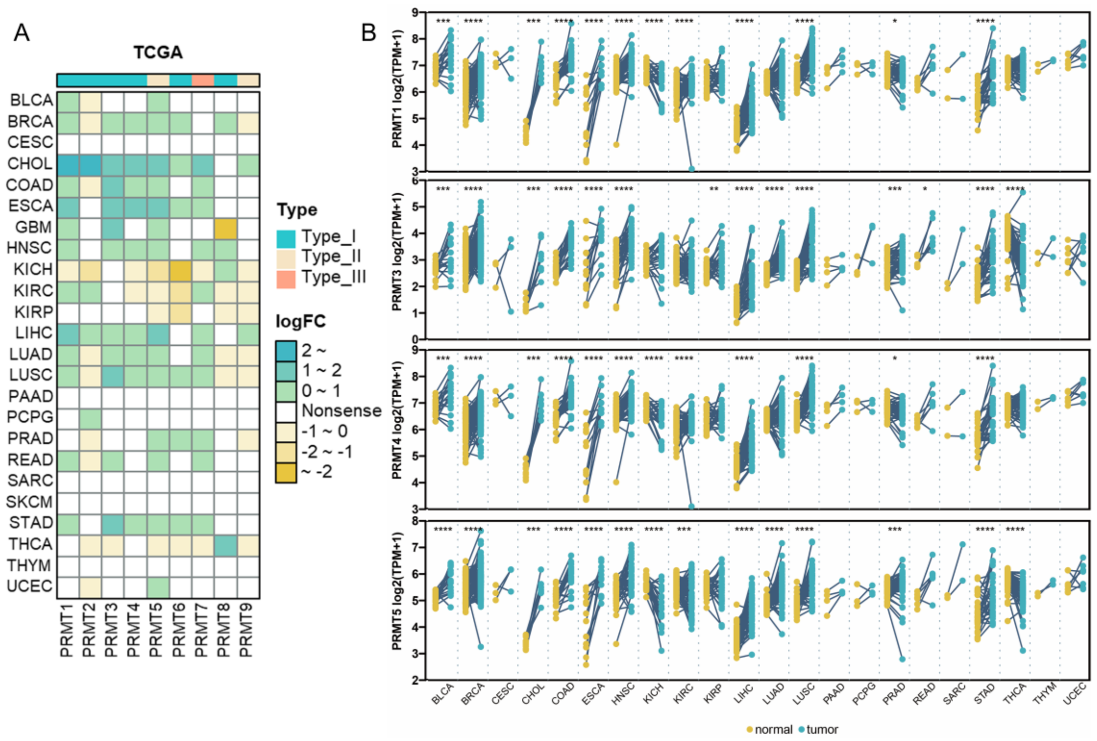

Figure S1: Expression profiles and subcellular localization of PRMTs.

(A) Differential mRNA expression of PRMT family members across TCGA pan-cancer types. Fold change and *P* values were determined by comparing tumor tissues with unpaired normal controls. Colors represent fold change, and "nonsense" indicates $P > 0.05$.

(B) Expression analysis of PRMT family genes in paired tumor and adjacent normal tissues across TCGA pan-cancer.

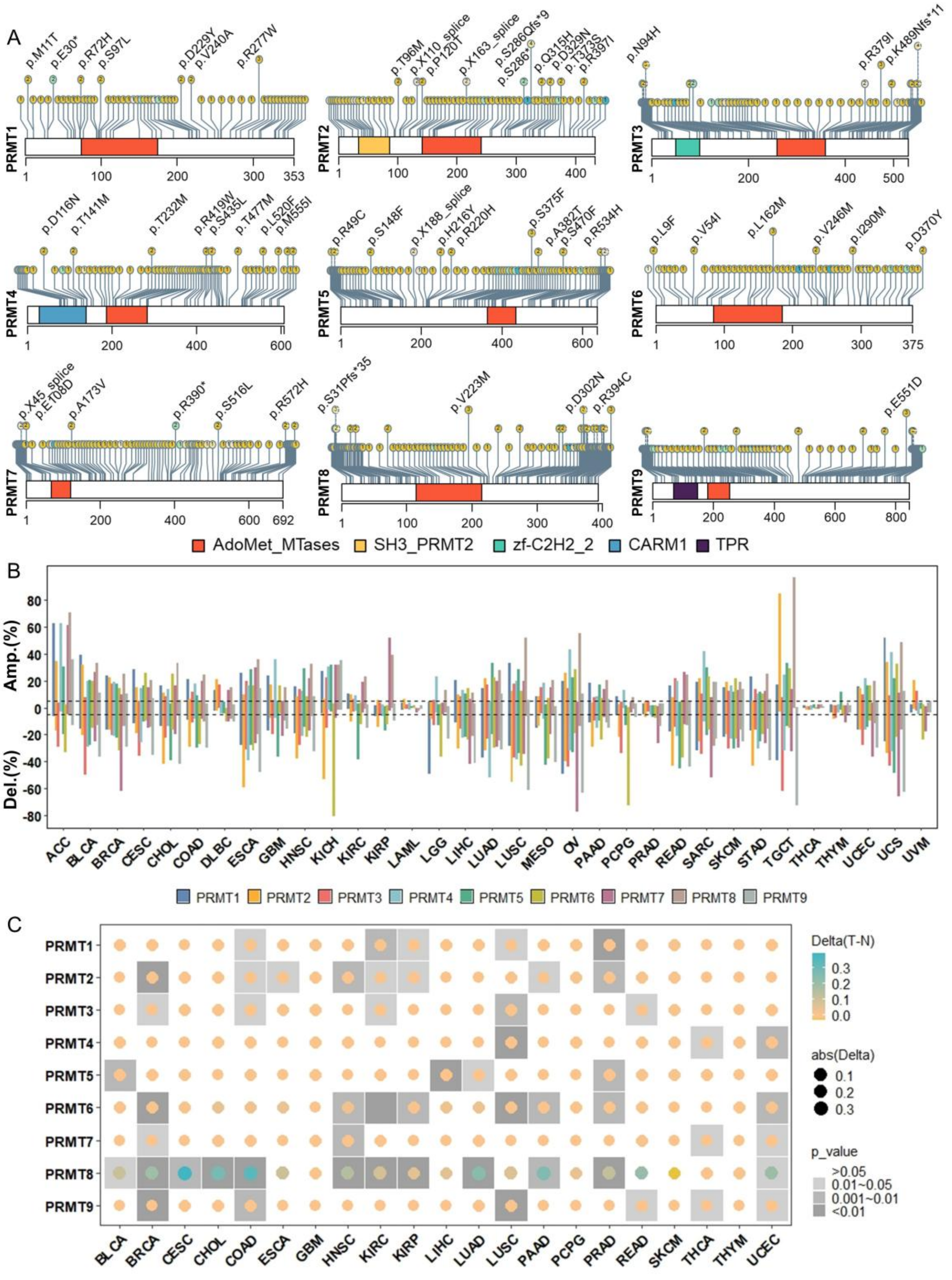

Figure S2: Genetic and epigenetic landscape of PRMTs across pan-cancer.

(A) Lollipop plots depicting the distribution and abundance of mutations within the protein domains of PRMTs.

(B) Frequency of CNV alterations of PRMT family members across various cancer types.

(C) Bubble plot showing the differential DNA methylation status of PRMTs between tumor and normal tissues. Circle size corresponds to the absolute difference in mean beta values, while circle color reflects the mean beta value in tumors compared to normal tissues. The significance of the

difference (*P* value) is indicated by the box color.

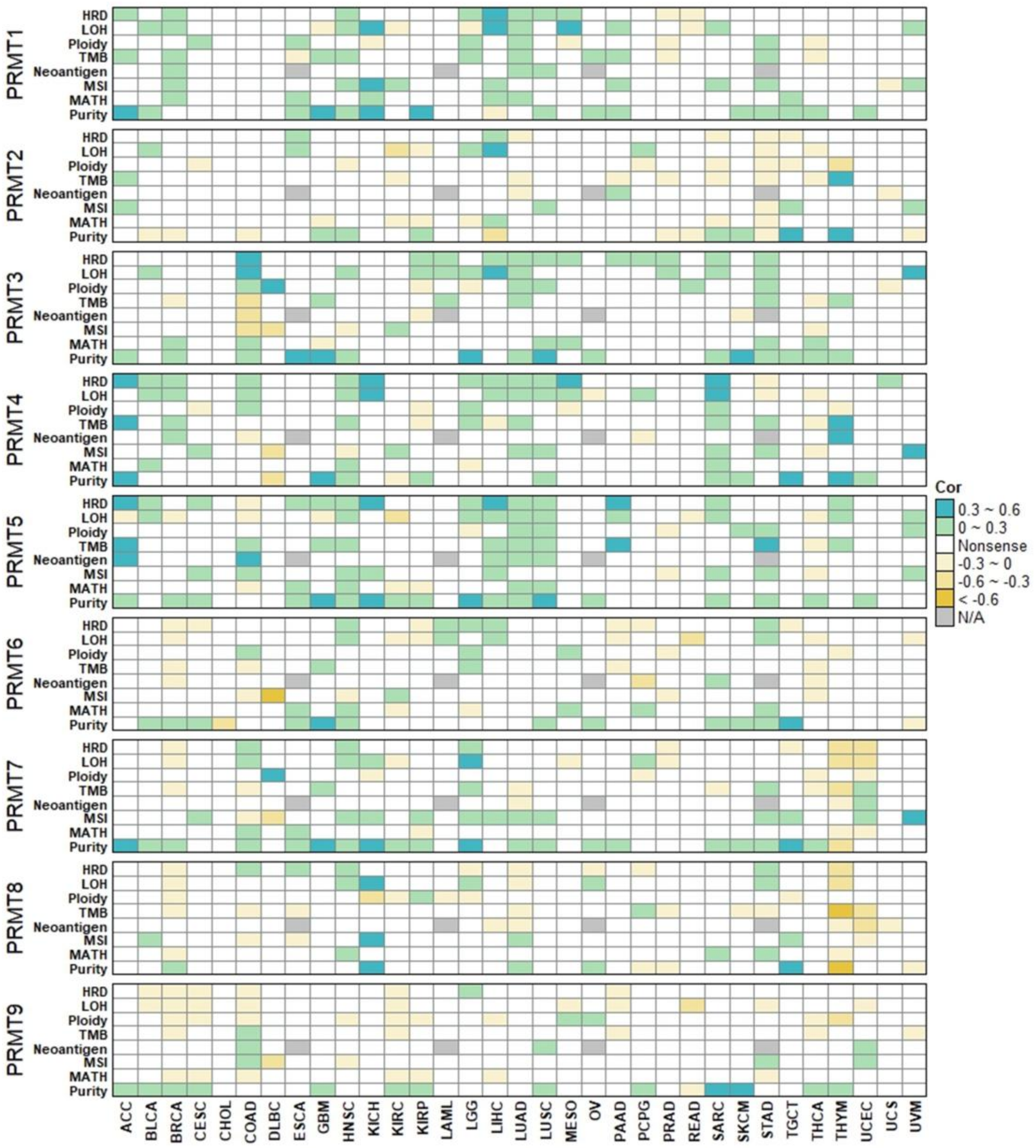

Figure S3: Correlation between PRMTs and genomic heterogeneity across pan-cancer. The color represents the correlation coefficient, "nonsense" indicates P > 0.05, and the N/A value represents the absence.

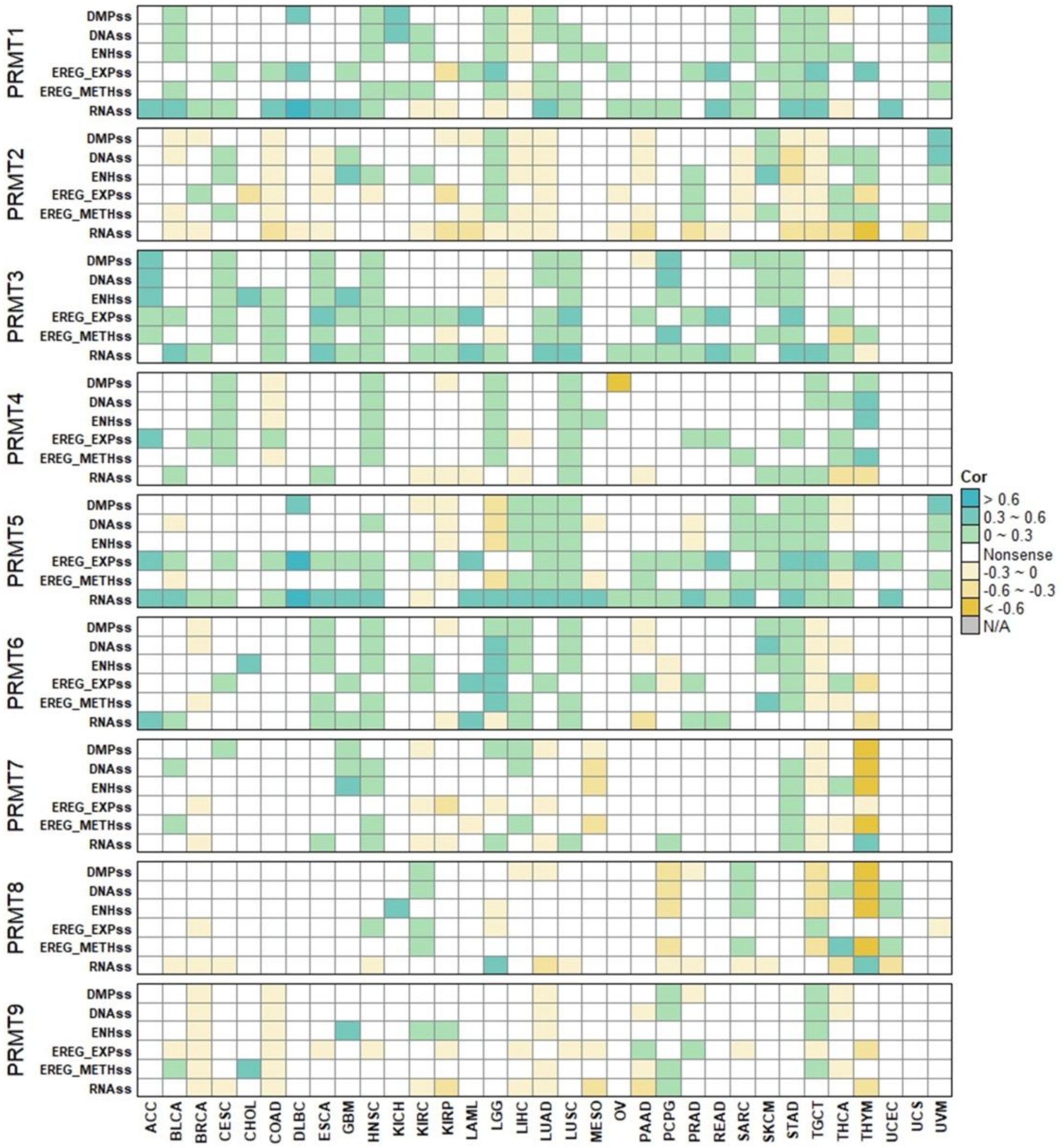

Figure S4: Correlation between PRMTs and tumor stemness indices across pan-cancer.

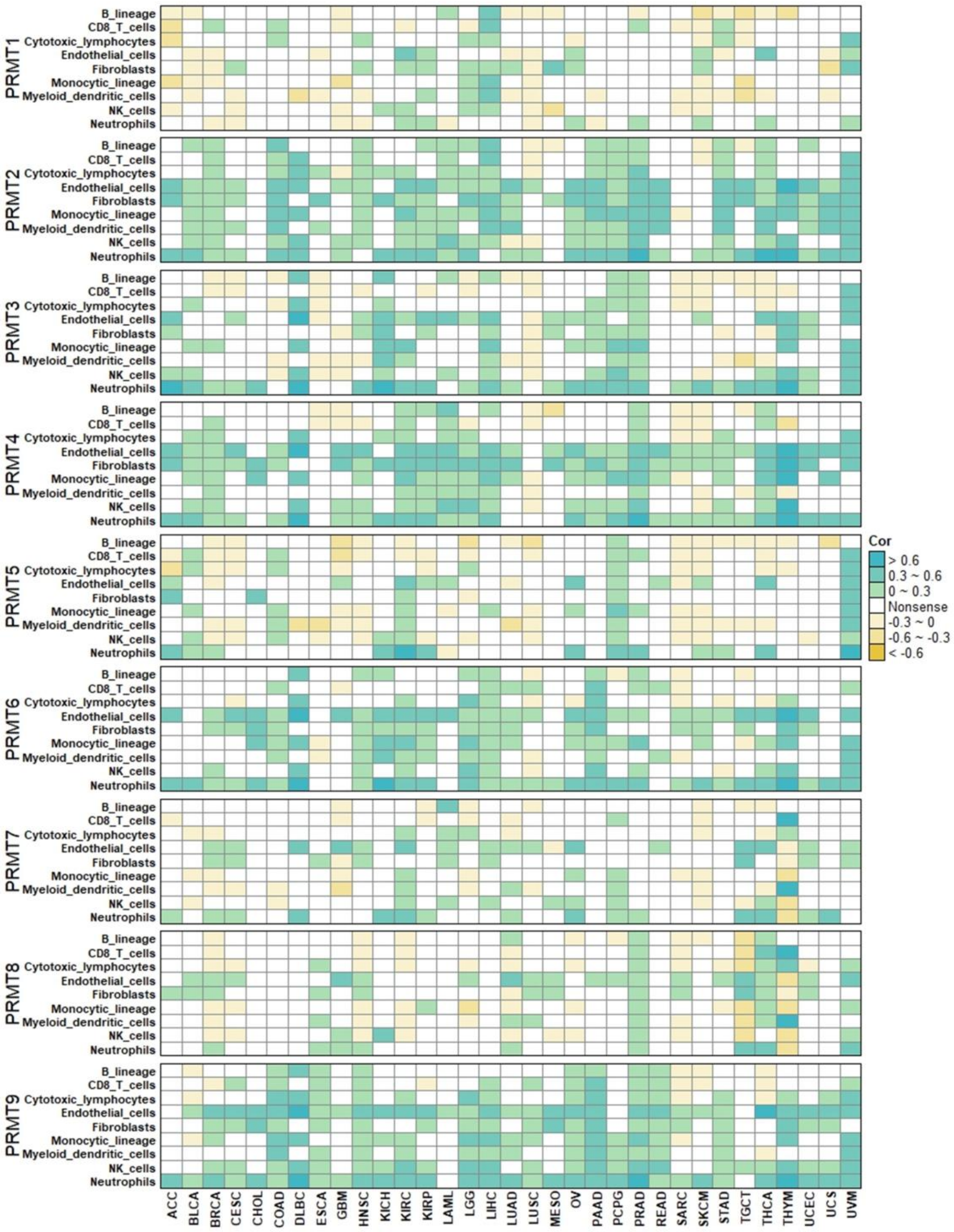

Figure S5: Correlation between PRMTs and immune cell infiltration across a broad range of cancer types.

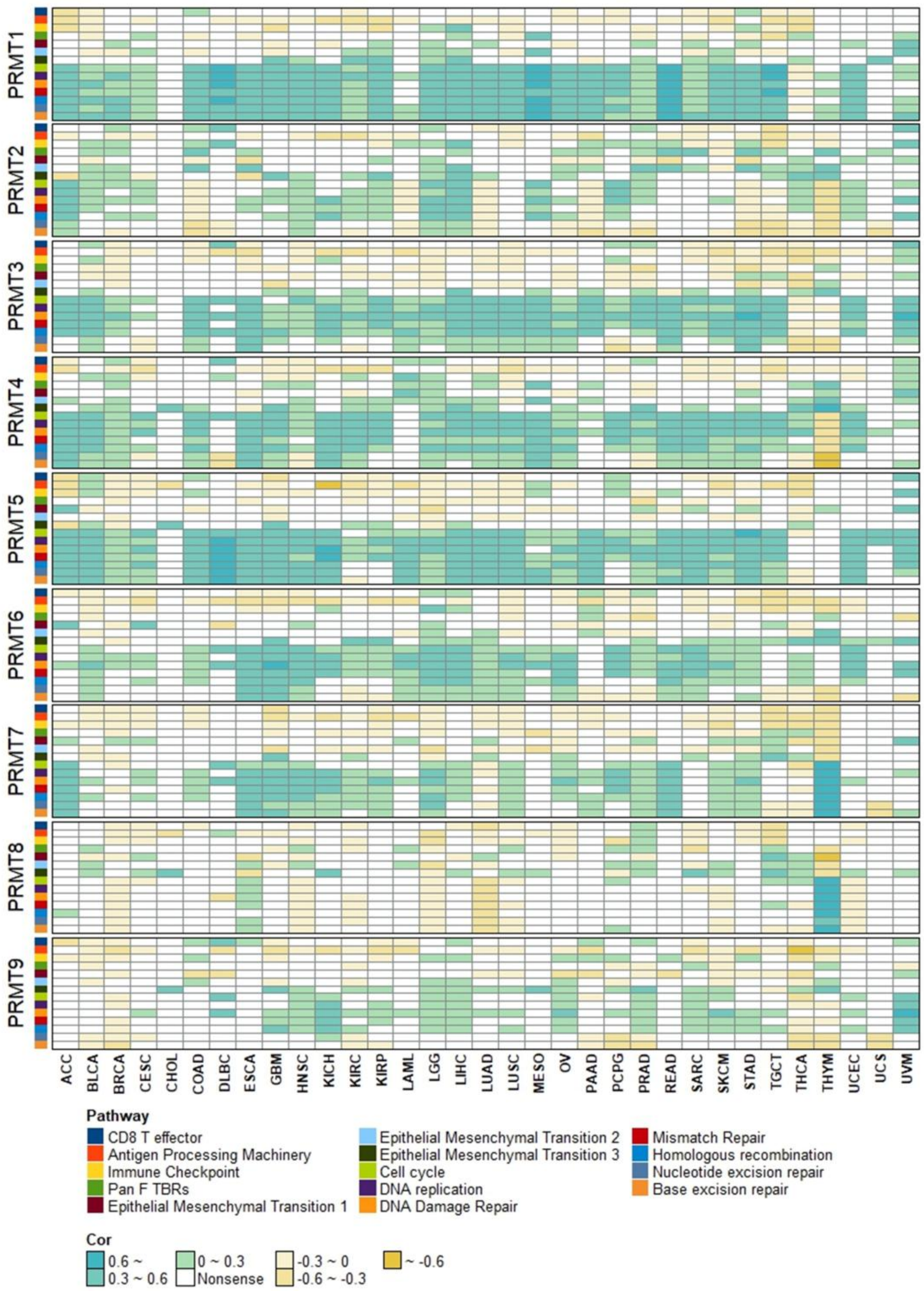

Figure S6: Correlation between PRMTs and signatures of immune activation, stromal remodeling, cell-cycle progression, and DDR pathways.

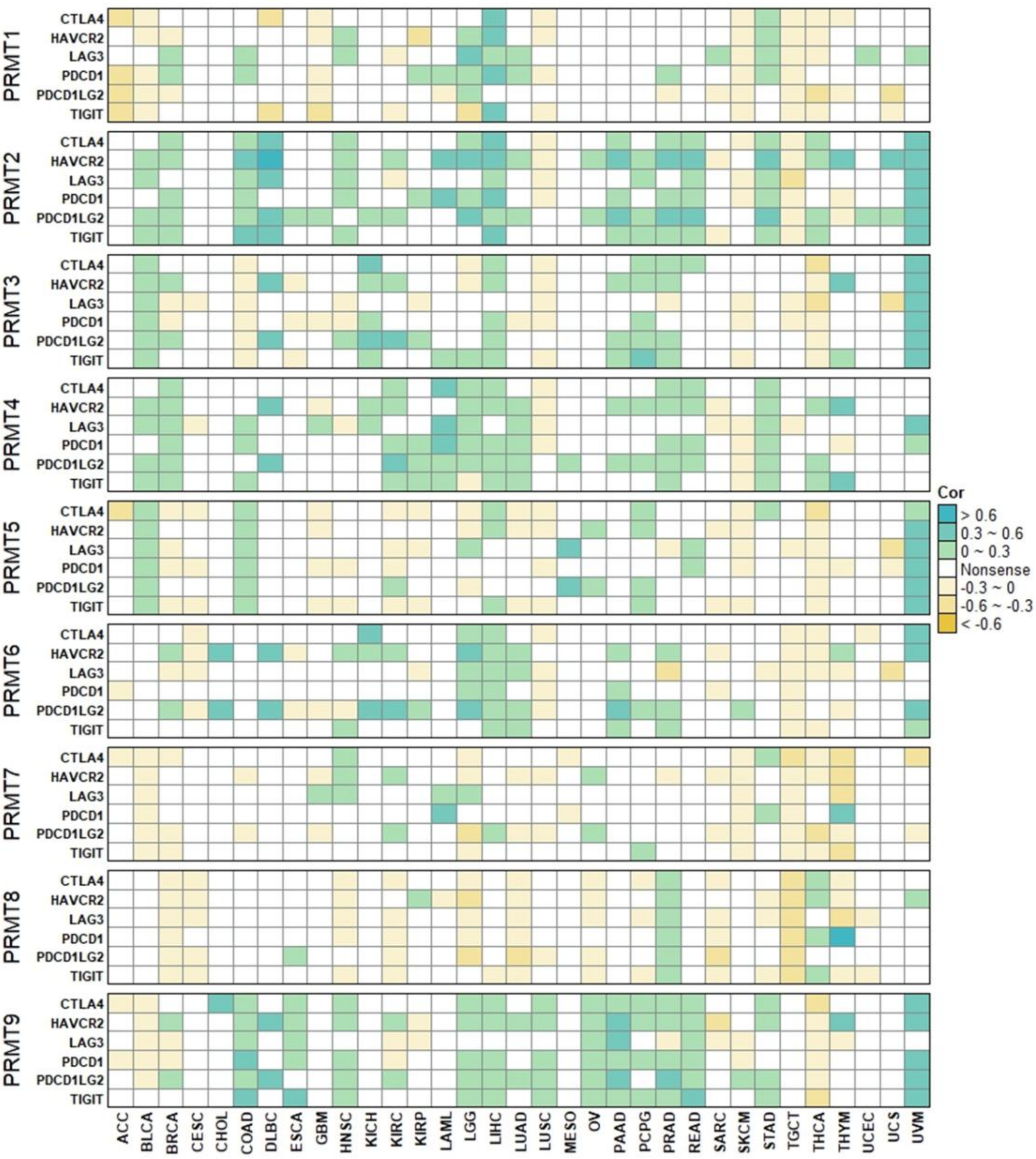

Figure S7: Correlation between PRMTs and immune checkpoint molecules across a broad range of cancer types.

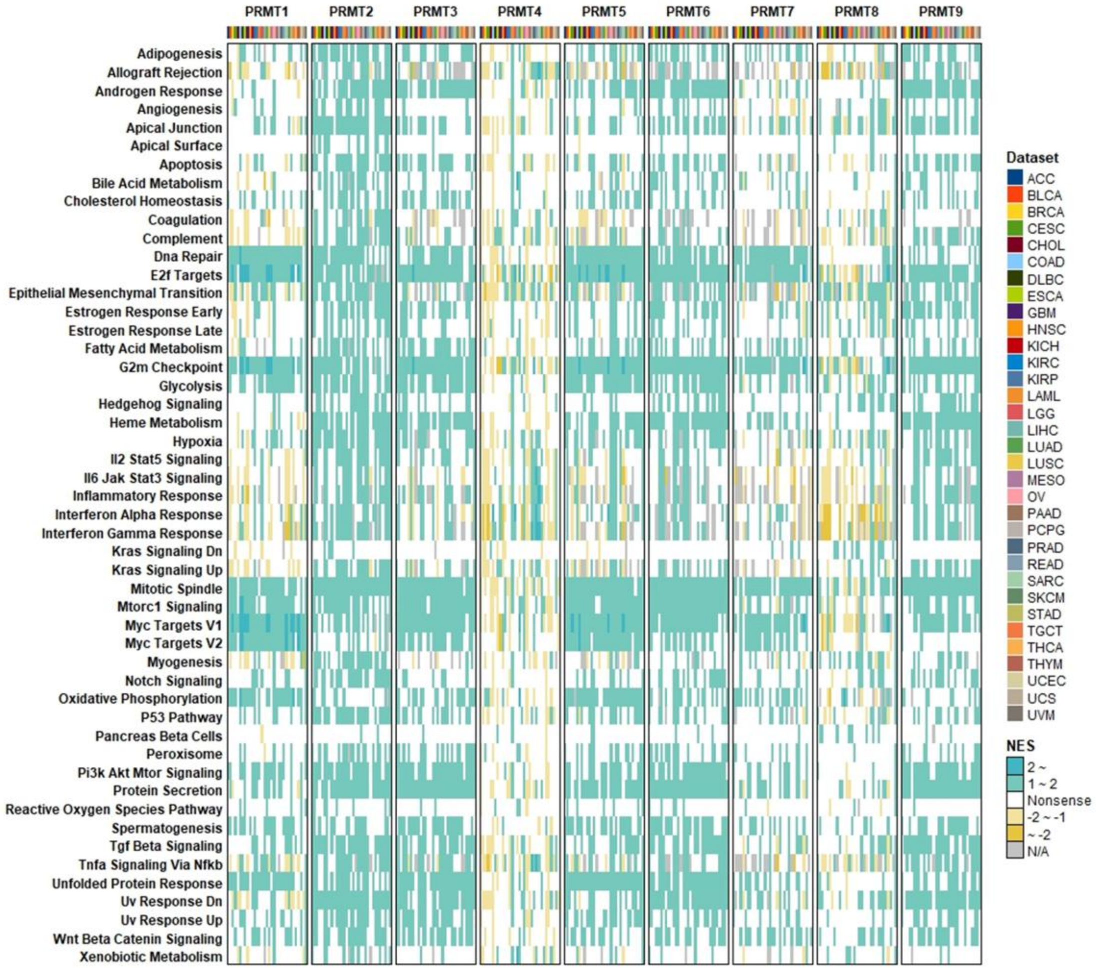

Figure S8: Associations between PRMTs and Hallmark Pathways across Pan-Cancer. Colors represent the NES for each pathway, and "Nonsense" indicates $P > 0.05$.

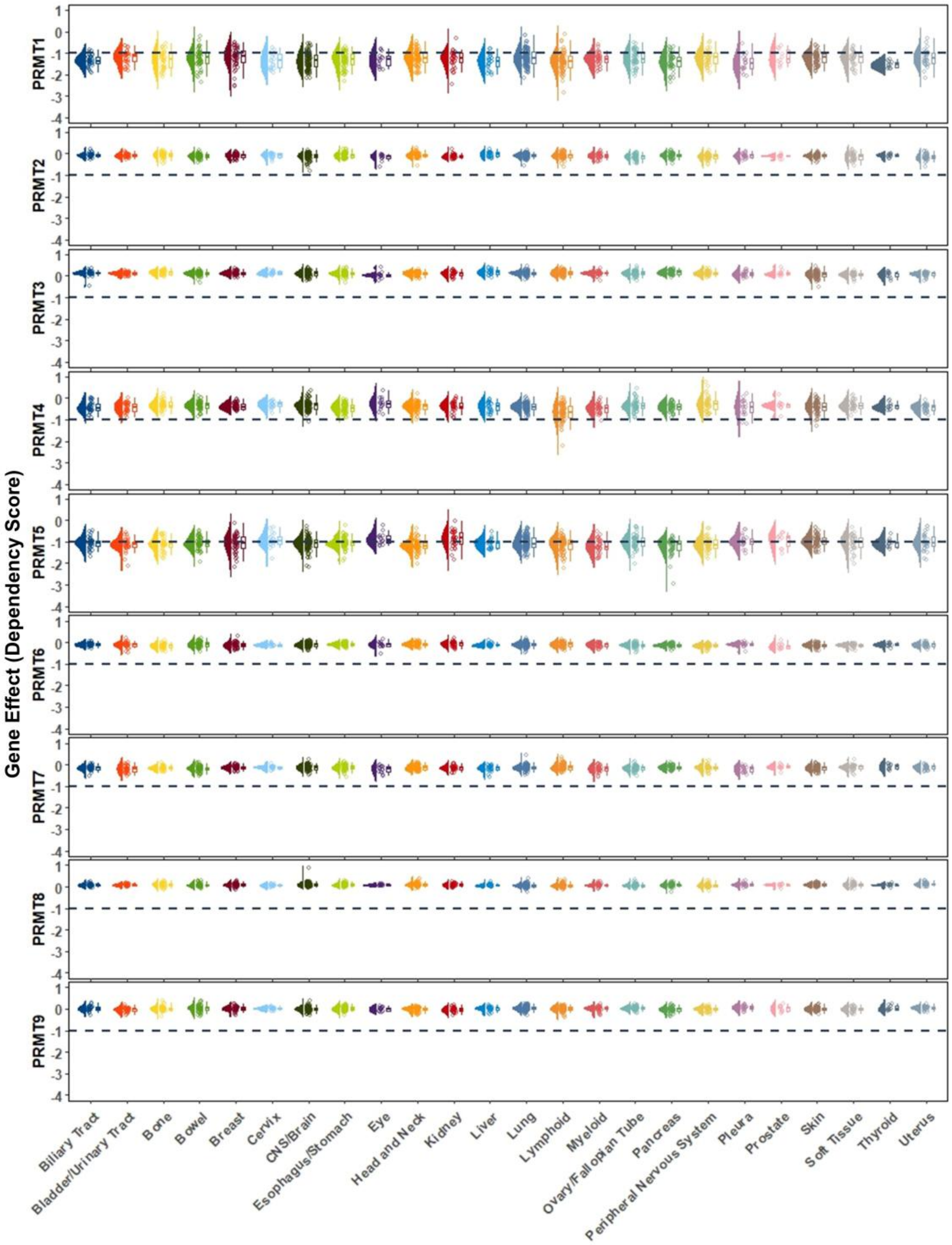

Figure S9: Evaluation of cell viability and gene dependency following PRMT knockout in the DepMap portal.

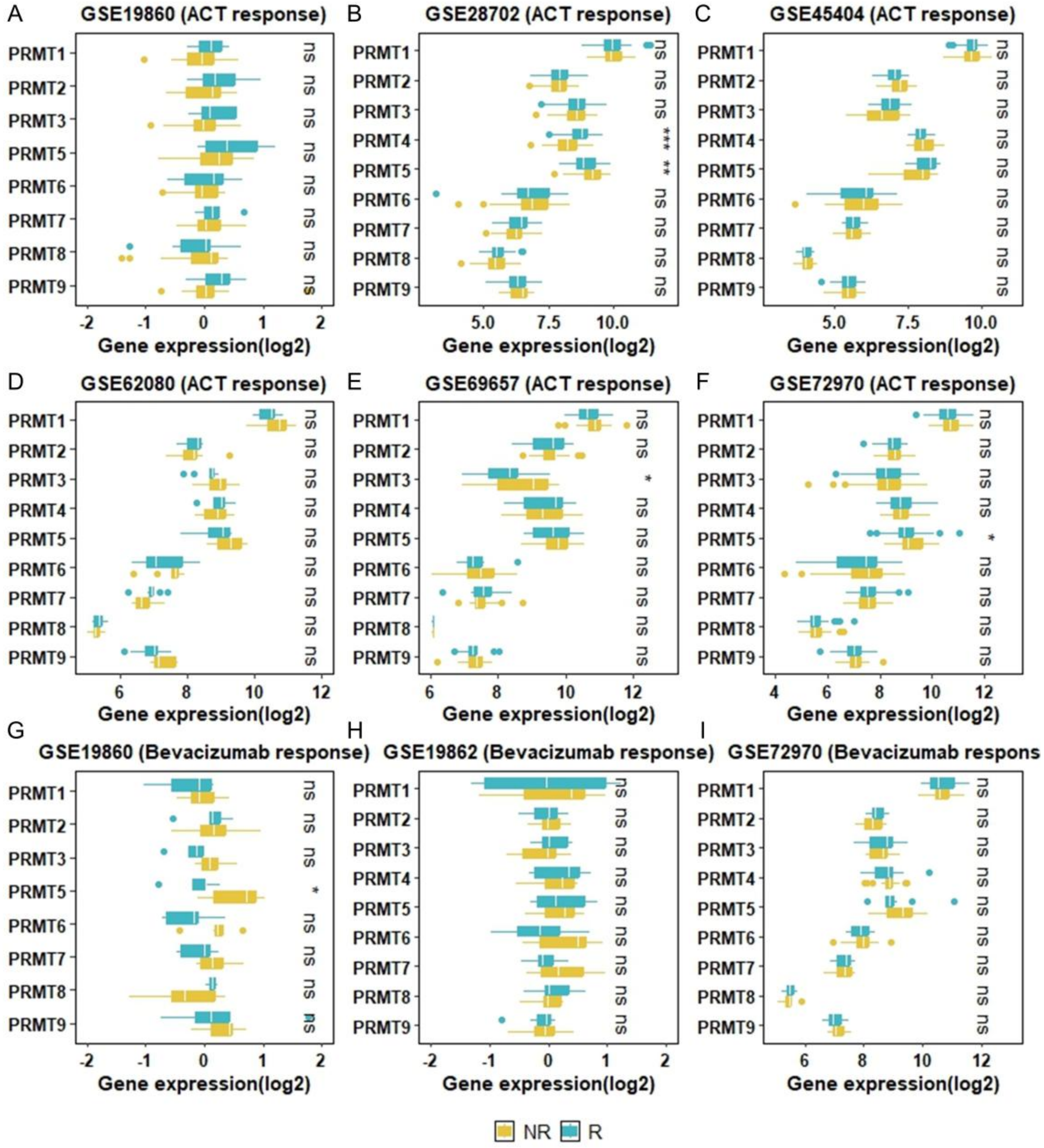

Figure S10: Association of PRMT expression with therapeutic response to 5-FU-based ACT and bevacizumab.

(A-F) Box plots showing PRMT expression in responders versus non-responders to fluorouracil-based ACT in GSE19860 (A), GSE28702 (B), GSE45404 (C), GSE62080 (D), GSE69657 (E), and GSE72970 (F). Note: PRMT4 probe was not available in GSE19860.

(G-I) Box plots showing PRMT expression in responders versus non-responders to fluorouracil-based ACT combined with bevacizumab treatment in GSE19860 (G), GSE19862 (H), and GSE72970 (I).

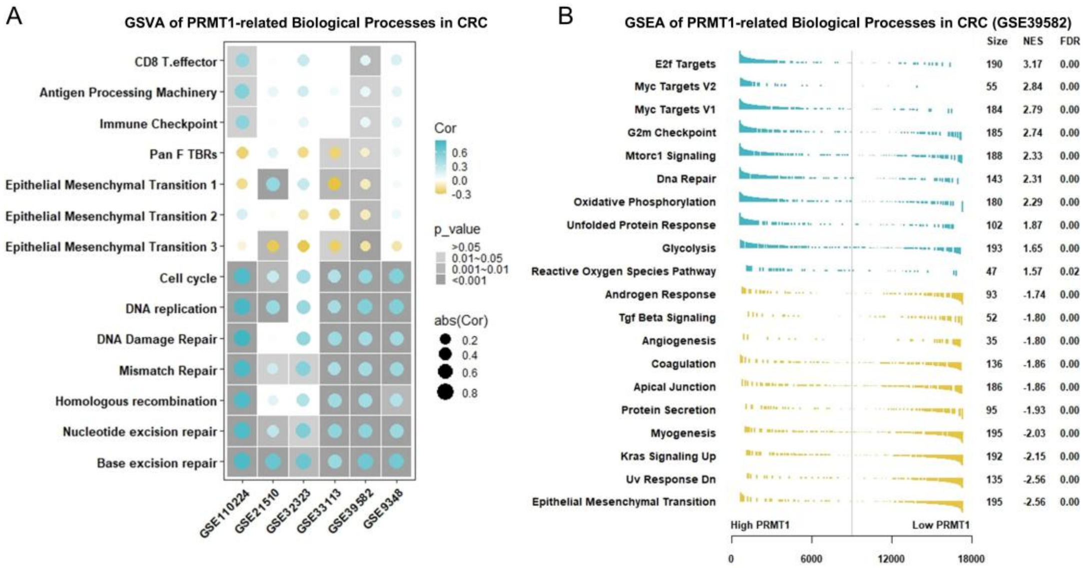

Figure S11. Functional enrichment and pathway analysis of PRMT1 across GEO datasets.

(A) Association of PRMT expression with GSVA-derived signatures of immune activation, stromal remodeling, cell-cycle, and DDR pathways across GEO cohorts. Circle size and color represent the absolute correlation coefficient and the correlation value, respectively, while the background color of each cell indicates statistical significance (P value).

(B) GSEA analysis results displaying the top 10 positively and bottom 10 negatively ranked signaling pathways based on NES.
